# The Pharmacopœia of the United States of America, 1820

**Published:** 1821-12

**Authors:** 


### CRITICAL ANALYSIS

### OF

### RECENT PUBLICATIONS, IN THE DIFFERENT BRANCHES OF MEDICINE AND SURGERY,

### In the Literature of Foreign Nations.

Πατρίδες ἄρα
Ανδράσιν, ὧ πατραῖς ἄνδρες, ἀγαλλόμεθα.

*The Pharmacopœia of the United States of America*, 1820. *By the Authority of the Medical Societies and Colleges.* 8vo. pp. 272. Ewer, Boston.

IN presenting to the English public an account of foreign publications, there are two courses which an Editor of a periodical work may follow, with equal advantage. He may either give a simple analysis of the foreign work with which he is desirous to make his readers acquainted, and nothing more; or he may do this, and at the same time inform them of what the critics of the nation to which the work itself belongs think of its merits and composition. This latter course is precisely what we determined to adopt in the case of the "National Pharmacopœia," as it is emphatically styled, of the United States of America. Deprived of the book itself, notwithstanding the pressing order to our correspondent to transmit it to us as soon as published,—and unable, consequently, to give an account of it,—we have deemed it best to lay before our readers a relation of the curious mode in which this compilation has been formed, and the opinion that is entertained respecting it in America, taken from one of their own Medical Journals. We have preferred, in fact, to let the Americans speak for themselves.

---

"THE work now before us may be considered as forming an era in the history of the medical profession. It is the first performance of the kind, as far as we recollect, compiled by the authority of the faculty throughout a nation. Collections of approved receipts for medicines have often been made in other countries, and there are several respectable ones in our own; but none has appeared under the broad and impressive sanction which distinguishes this. Sometimes individual professors and lecturers have published results of their investigations. The Materia Medica of BOERHAAVE, the Apparatus Medicaminum of MURRAY, and the Formulæ Medicamentorum of HUGH SMITH, are examples of this kind. Occasionally practical physicians have reduced to writing their knowledge and experience. The Processus Integri of SYDENHAM, the Pharmacopœiæ of BERKENHAUT and DALE, and the Dispensatories of QUINCY and JAMES, belong to this class. A more solemn and authoritative body of instruction has proceeded from certain colleges of physicians, such as those of the learned associations of London, Edinburgh, and Dublin; and, latterly, France has furnished, by command of her monarch, a national Pharmacopœia, under the title of "Codex Medicamentarius." It remained for the people of America to frame a work of this kind, which should emanate from the profession itself; which should be founded upon the principles of representation; and embody, as nearly as possible, the whole *corpus medicum* in these free, independent, and united States.

"A political constitution had been prepared by a convention assembled for that purpose; and it was conceived that a convention of delegates, if duly appointed by the several colleges and societies, might form a medical constitution for the use of all who prescribed, and of all who consumed, medicines.

"The Historical Introduction contains a summary, derived from the original documents in the hands of the corresponding secretary, of the commencement, progress, and completion, of this Pharmacopœia. (P. 5—16.) It conveys curious and interesting information. By this it appears that the project was first offered to the New-York County Medical Societies, early in 1817, by LYMAN SPALDING, M.D. and did receive their sanction. It was soon after adopted by the State Society in Albany, where measures were promptly taken to carry it into effect. After making known the plan to other individuals and associations, by regular correspondence, the concurrence of professional men was formally and solemnly given from the following states; to wit,—Vermont, New-Hampshire, Massachusetts, Rhode-Island, Connecticut, New-York, New-Jersey, Pennsylvania, Delaware, Maryland, Kentucky, Ohio, Indiana, South Carolina, Georgia, New-Orleans, and the district of Columbia.

"Pursuant to the original design, a national convention was held at the city of Washington, in January 1820; and this is the result of their labours. It appears, by a certificate of the president, Samuel L. Mitchill, that Lyman Spalding of New-York, Thomas H. Hewson of Philadelphia, Eli Ives of New-

Haven, Elisha De Butts of Baltimore, and Jacob Bigelow of Boston, were the preparing or publishing committee.

"In a sensible and well-written Preface, the principles which governed the convention and the committee are laid down; the difficulties of selecting a Materia Medica, and a body of officinal compounds, from the enormous mass of known articles, particularly stated; the delicate scholarship requisite to the formation of a nomenclature, is portrayed; and the compilers seem to be fully aware of the profound knowledge of botany and chemistry that are necessary for the execution of so arduous a task. They have had the great European models before them; and, if they have not made a larger work, or a different work, it was not because they wanted precedents and receipts, for these were in amount cumbrous and embarrassing; but because they chose to render their performance as plain and simple as the state of society, and the state of science, seemed to demand.

"The Pharmacopœia is published both in Latin and English. The reason for this arises partly from the dignity of the learned profession to which the members of the convention belong; and partly from the consideration that, in various parts of our land, especially in the French and German settlements, the ancient language would be more intelligible than the vernacular.

"That the convention understood the pharmaceutical bearing of the investigation which they foresaw their performance would undergo, plainly appears from the following extract:

"It is the object of a Pharmacopœia to select, from among substances which possess medicinal power, those, the utility of which is most fully established and best understood; and to form from them preparations and compositions, in which their powers may be exerted to the greatest advantage. It should likewise distinguish those articles by convenient and definite names, such as may prevent trouble or uncertainty in the intercourse of physicians and apothecaries.

"The value of a Pharmacopœia depends upon the fidelity with which it conforms to the best state of medical knowledge of the day. Its usefulness depends upon the sanction it receives from the medical community and the public, and the extent to which it governs the language and practice of those for whose use it is intended.

"In most European countries, works of this kind have appeared under the authority of medical colleges and corporations. Their usefulness has generally been co-extensive with the influence of the bodies of men from whom they have originated. If they have been less useful than might have been hoped from their character and objects, it is because different works of this kind, proceeding from different sources, and disagreeing with each other in their details, have been permitted to circulate in the same community; thus interfering with each other, and frequently introducing confusion into the practice they were intended to regulate.

"In the United States, the evil of irregularity and uncertainty in the preparation of medicines has been felt with peculiar weight. Besides a number of Pharmacopœias and Dispensatories founded upon them, which have been produced in different parts of the Union, we import various foreign works of the kind, some of which have become naturalized by re-publication in the country. The druggist and the medical practitioner are supplied, as their convenience may direct, with any one or more of these books; and, of course, the character of medicinal preparations is liable to vary in every state and city of the Union; and the physician is exposed, unconsciously, to administer to his patient medicines essentially different from those which his judgment has prescribed.

"That this evil has not been earlier remedied, is to be attributed, not so much to a want of conviction, on the part of the medical faculty, of the importance of the subject, as to the difficulty of obtaining in such a work the general co-operation of physicians throughout a country so extensive as ours. In several of the states, measures had been taken by the faculty to regulate the preparation of medicines, and with success, as far as it respected the circle of their respective practice.* But a national Pharmacopœia, which should be established and adopted by the consent of all the medical corporate bodies throughout the United States still remained a great desideratum; being evidently the only mode by which a uniform system could be introduced at once in all parts of the American territory. In the present volume, a work of this kind has for the first time been undertaken; and, after being gradually matured by the advice, consent, and co-operation of physicians in all parts of the Union, it is at length committed to the press, as the result of their deliberations and decisions.

"In the formation of the American Pharmacopœia, the general convention, and their publishing committee, have had to encounter those difficulties which must always attend the first publication of works of this kind. The selection of a materia medica,—the formation or adoption of preparations and compounds,—and the establishment of a pharmaceutical nomenclature,—have constituted their chief labour. On each of these departments of the work they have endeavoured to bestow that degree of careful inquiry and mature deliberation which the importance of the occasion demanded; and have pursued the course which appeared to them best suited to supply the wants, and promote the interests, of the medical community in all sections of the country.

"The fault of the lists of the materia medica which have been adopted in different countries, has always been their redundancy rather than their deficiency. The number of articles necessary for the management of diseases, and especially of those which any indi-

---

* The Pharmacopœia of the Massachusetts Medical Society was published in 1808, and afterwards adopted by the Medical Society of New-Hampshire. The Pharmacopœia of the New-York Hospital was published in 1816.

vidual physician actually employs, is always very far short of the catalogue afforded by most Pharmacopœias. Besides, as the progress of medical discovery continually tends to the introduction of new articles into use, the materia medica must soon grow to an unmanageable size, if its enlargement be not followed by a corresponding retrenchment of superfluities. In consequence of reasons of this sort, many articles contained in European books have been omitted in the American Pharmacopœia. These omissions have been made only where the articles in question were considered inert, or where they were abundantly superseded by substitutes more powerful and more accessible.

"The system of retrenchment might, no doubt, have been more rigorously exercised without ultimate disadvantage to the interests of medicine: but it was thought to be at present more conducive to the public good to retain on the list all those medicines which were believed to be so much in use in any part of the United States, that their omission would occasion inconvenience to physicians and apothecaries, and render the book less applicable to their wants.

"In regard to indigenous vegetables, a considerable number, no doubt, possess important and useful properties; others have pretensions not yet fully settled. But, as it happens in most countries, the number of simples occasionally employed in practice is much greater than it suits the proper compass of a Pharmacopœia to contain. In the present work, those native articles have been introduced which were considered to possess qualities sufficiently important, or which were found to be so much employed by practitioners, as to give them any claim to the character of standard medicines. In several instances native plants have been substituted for European ones of the same genus, where their qualities were esteemed the same.

"With a view of discriminating between articles of decided reputation or general use, and those the claims of which are of more uncertain kind, the convention determined to refer to a secondary list such substances as were deemed of secondary or doubtful efficacy, retaining only on the principal list articles which might be considered of standard character. In the execution of this measure, particularly in the case of new medicines, they have possibly consigned to the secondary list some articles of more efficacy than others which they have retained on the primary ones. In doubtful cases they have preferred to swell the subordinate rather than the primary catalogue, especially as this arrangement will be most likely to prompt farther investigations into the character of the substances in question.

"In that part of the work which contains the formulæ for the preparations and compositions, the convention have preserved those only which have received the sanction of the faculty in this country or in Europe. They have thought it their duty to insert all which were reported to them by the district conventions, except in cases where the near similarity of two preparations has rendered one of them superfluous. Alterations of established formulæ have been avoided, unless it be where the convenience and simplicity of medicines could be promoted without changing their operation or activity."

That the convention also knew that their work was not in all respects complete or wholly free from faults, appears from the foresight shown in relation to a future revision and republication of the work. The convention understood that *incuriæ* or over-sights might be expected, and that discoveries would continue to be made; they, therefore, before their adjournment, made the following provision for the future revision and republication of the book, that all errors might be corrected, and all improvement incorporated.

"Resolved, That in case of the death, resignation, or inability to act, of the president of this convention, the secretary shall forthwith issue writs of election to the several delegates of this convention, who, by written ballots addressed to him, may elect another president.

"Resolved, That, in case of the death, &c. of the secretary, the president shall cause another to be elected, as above described.

"Resolved, That the president of this convention shall, on the 1st of January, 1828, issue writs of election to the several incorporated state medical societies, &c. in the northern district, requiring them to ballot for three delegates to a general convention, to be held at Washington on the 1st of January, 1830, for the purpose of revising the American Pharmacopœia; and that these several institutions be requested to forward to the president, on or before the 1st day of April, 1829, the names of three persons thus designated by ballot; and the president of the convention is hereby requested, on the said day, to assort and count the said votes, and to notify the three persons who shall have the greatest number of votes of their election: and, in case there should not be three persons who have a greater number of votes than others, then the said president is desired to put a ballot into the box for each of those persons who have an equal number of votes, and draw therefrom such number of ballots as shall make the number of delegates three, and notify as before.

"This resolution to apply, in like manner, to the middle, southern, and western districts.

"In case neither of the delegates from a district can attend, it shall be the duty of such delegates to appoint a substitute who can attend.

"Whereas the progressive improvements in medicine, as well as other causes, may render it expedient to revise the Pharmacopœia at an earlier period than is expressed above, it shall be the duty of the president to call the attention of the medical societies and colleges to the subject, whenever, in his opinion, the public good may require it."

The performance consists, as is usual in compilations of this kind, of a materia medica, and a body of officinal formulæ, carefully selected from the best authorities, and adapted to the practice of medicine in the United States. To the catalogue of the former is annexed a supplementary list of articles, too well recommended to be rejected, and worthy of further trial by judicious practitioners. (P. 51—57.) The latter are arranged under the several heads of Vinegars, Acids, Ethers, Alcohol, Gum Ammoniac, Antimony, Waters, Silver, Gold, Arsenic, Barytes, Bismuth, Lime, Cerates, Collyriums, Confections, Copper, Decoctions, Plasters, Extracts, Iron, Quicksilver, Infusions, Liniments, Magnesia, Honeys, Mixtures, Oils, Pills, Lead, Potash, Powders, Soda, Spirits, Sponges, Tin, Sulphur, Syrups, Troches, Tinctures, Ointments, Wines, and Zinc.

Our limits forbid us to go into a detailed examination of the manner in which the convention has executed the arduous and difficult task imposed upon them. It is sufficient for the present to observe, that, as the great object was to produce, as far as possible, uniformity throughout our populous and increasing country in the preparation of medicines, and thereby to produce additional confidence of patients in their physicians and prescribers, the faculty will receive this Pharmacopœia as the result of their own spontaneous efforts to promote the good of their fellow citizens, and to add dignity to the medical profession.

After all this, it may nevertheless be expected that differences of opinion will exist. Authors and venders interested in the copyrights of books now before the public will probably not be pleased with the introduction of this volume: apothecaries, accustomed to prepare medicines according to the receipts and forms in such works, may quit them with reluctance, or refuse to relinquish their habits of business. Physicians educated in Europe, and their pupils and imitators in America, may feel inclined, and even determined, to make preparations and prescriptions according to the dominant Pharmacopœias of the Colleges or Schools in which they were educated; and chemists may come forward in this case, as they have done in regard to the Pharmacopœiæ of the London and other colleges, and show abundance of their science in detecting alledged errors in the combination of simples here published. All this, we understand, was foreseen by the convention; but, while the right of extemporaneous prescription is pursued, and the present work comes forth as a guide and rule only for the simples and officinal compounds, we trust it will be cordially received by the profession generally."

---

Another American critic does not treat so leniently this performance of the medical convention. He goes boldly to work* in surveying their production, and exposes its various defects, errors, and incongruities. This critic is particularly angry

* *The Philadelphia Journal*, for August 1821, p. 367.

against the *convention* for having presumed to sell the copyright of a work, to which no individual in the nation can lay exclusive claim; since, without a power ceded to that effect to the members of the convention collectively, the Pharmacopœia, being published by a set of *unincorporated* men, is the *bona fide* property of any of the citizens of the Union. A disposition is even announced, on the part of a member of the profession, to try the case in a court of law, forthwith.

But the most singular part of this *second* review is the concluding paragraph, which, for the sake of those who take an interest in American literature, we have taken the liberty to transcribe.

"We recommend to every physician and apothecary in the United States, to consider well the merits of a work which professes to claim the high rank of Pharmacopœia of the United States. It is essential to the character of the nation at large,—it is more peculiarly so to the medical profession,—that its real value be properly appreciated; since we may assert, with truth, that every person must be interested in a book which may be said to contain the issues of life and death. If, upon thoroughly investigating its contents, it be approved of, let it universally be upheld: if defects are observed, let them, by the call of another convention, be speedily rectified; and, in the mean time, let the members of the last convention be required most assiduously to buy up the whole impression, that not a copy may reach the European shores. If this be not done, we here protest, on behalf of our medical brethren throughout the Union, against the misnomer by which this work is denominated the Pharmacopœia of the United States, and will cheerfully unite in any proper measure by which it may be speedily set aside."

